# Echocardiography as a Useful Tool for Differentiating Acute Pulmonary Embolism From Acute Coronary Syndrome: A Case Report

**DOI:** 10.7759/cureus.71643

**Published:** 2024-10-16

**Authors:** Varshitha T Panduranga, Ammar Y Abdulfattah, Victor F de Souza, Adam S Budzikowski, Samy I McFarlane, Sabu John

**Affiliations:** 1 Department of Internal Medicine, State University of New York Downstate Medical Center, Brooklyn, USA; 2 Department of Cardiology, State University of New York Downstate Medical Center, Brooklyn, USA

**Keywords:** acute coronary syndrome, echocardiography, mcconnell’s sign, pulmonary embolism, pulmonary hypertension

## Abstract

Both acute coronary syndrome (ACS) and pulmonary embolism (PE) are life-threatening medical emergencies with overlapping symptoms and laboratory findings. Differentiating these two emergencies and initiating proper treatment are of paramount importance for good outcomes. In this report, we present the case of a 60-year-old male with a history of seizure disorder and hyperlipidemia, who presented to the emergency department (ED) after a syncopal episode preceded by three days of brief episodes of chest pain. In the ED, the initial electrocardiogram (EKG) showed normal sinus rhythm with T wave inversions in the anterior leads, and elevated high-sensitivity troponin levels peaked at 58 ng/mL before declining to 38 ng/mL. Elevated lactic acid and anion gap suggested a seizure, and the patient was discharged after lab tests and clinical status normalized. The patient returned the next day with recurrent syncope, and this time troponin levels were significantly elevated to 151 ng/mL, with a pro-BNP (brain natriuretic peptide) of 1,705 pg/mL. The patient was admitted with an initial diagnosis of ACS. The initial evaluation, including chest X-ray and EKG, was unremarkable. However, echocardiography revealed an interesting finding of right ventricular free wall akinesia with sparing of the apex-McConnell’s sign-suggestive of PE, which significantly changed the diagnostic approach. PE was later confirmed by computed tomography angiography. This case highlights the critical role of echocardiography in distinguishing PE from ACS, especially in emergency care settings in patients with atypical and rare presentations.

## Introduction

Pulmonary embolism (PE) is a form of venous thromboembolism (VTE) and is the third most common cause of cardiovascular death worldwide after strokes and acute myocardial infarctions (MIs). Acute coronary syndrome (ACS) and PE are both cardiovascular emergencies but differ significantly in their pathophysiology and clinical management. ACS arises from an acute reduction in coronary blood flow, usually due to plaque rupture or thrombosis within the coronary arteries, leading to myocardial ischemia and infarction. This often manifests as chest discomfort, radiation of pain, and EKG changes such as ST-segment elevation or depression, along with elevated cardiac biomarkers such as troponin. Conversely, PE is a consequence of thrombus migration from the venous system, typically from the deep veins of the lower extremities, into the pulmonary arterial circulation, leading to impaired pulmonary blood flow and subsequent right heart strain. Understanding these distinct mechanisms is crucial, as the treatment protocols differ dramatically. Diagnosis of PE can be challenging due to its non-specific nature of presentation.

The classic symptoms of PE include hypoxia, pleuritic chest pain, tachypnea, and tachycardia [1]. PE can present with syncope, or signs of right ventricular (RV) dysfunction, as well as elevated troponin levels due to RV ischemia from increased afterload [1]. However, PE appears to be a relatively rare cause of syncope in patients presenting to the emergency department (ED). In a study of 1,397 patients presenting with syncope to the ED, PE was identified in 19 (1.4%) cases [2]. Approximately 720,000 Americans suffer from MI or die from coronary artery disease annually [3]. In elderly patients, ACS often presents with vague and atypical symptoms, such as dyspnea, nausea, diaphoresis, and syncope, due to a range of comorbidities and accompanying complaints [4]. A study found that atypical presentations of ACS occur in 4.6% of cases [5]. Due to the potential for atypical presentations of both PE and ACS, echocardiography can act as a crucial tool for accurately diagnosing the underlying cause of these non-specific symptoms in acute settings. Timely diagnosis and treatment of these two critical conditions are essential, as delays can increase morbidity and mortality.

## Case presentation

**Figure 1:**
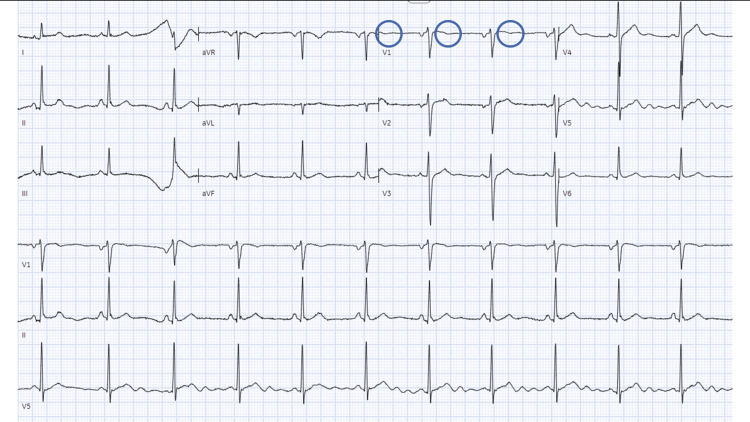
Electrocardiogram showing normal sinus rhythm at 68 beats per minute with T wave flattening in lead V1 (blue circles)

A 60-year-old male with a past medical history of seizure disorder on levetiracetam and hyperlipidemia on rosuvastatin presented to the ED after an episode of syncope. The patient felt lightheaded while walking up the stairs in his house and lost consciousness briefly. No tongue biting or urination occurred during the episode. The patient also complained of mid-chest pain that started around three days prior to this syncopal episode, which was atypical in nature. Initial vital signs and physical examination were unremarkable in the ED. An EKG was performed in the ED, which revealed normal sinus rhythm with T wave flattening in lead V1 (Figure 1). Initial high-sensitivity (HS)-troponin level was 58 ng/mL, and a basic metabolic panel showed an elevated anion gap of 19 with a creatinine of 1.27 mg/dL (with a baseline of 0.86 mg/dL), which indicated a likely seizure given the elevated anion gap secondary to lactic acidosis. He was given fluids, and repeated lab tests showed normalization of creatinine and anion gap and a downtrend of HS-troponin to 38 ng/mL. The patient refused to stay for observation for 24-48 hours to complete syncope work-up, and was discharged against medical advice with outpatient follow-up with neurology and cardiology.

**Figure 2:**
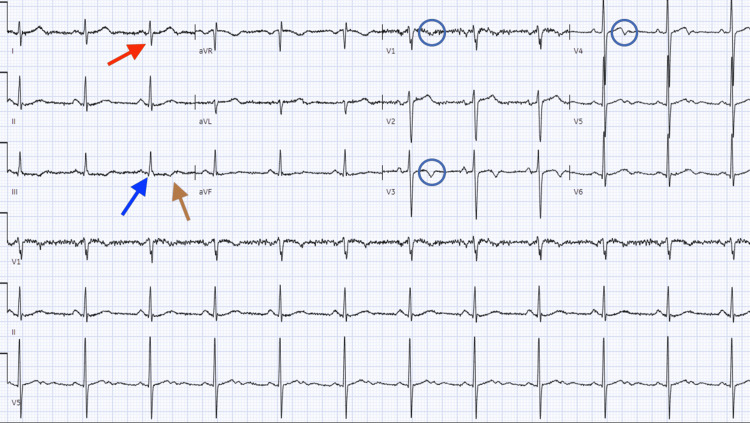
Electrocardiogram revealing normal sinus rhythm at 72 beats per minute with S1Q3T3 morphology and T-wave inversions in V1, V3, and V4 (blue circles) Red arrow indicates the S wave in lead I, blue arrow indicates the Q wave in lead III, and brown arrow indicates the inverted T wave in lead III.

The patient returned to the ED the next day following a similar episode of loss of consciousness. He started climbing the stairs and immediately felt lightheaded. He quickly sat down as he feared he would fall and then became briefly unresponsive. The episode was witnessed by his wife, who reported no tonic-clonic movements, urinary incontinence, tongue biting, head strike, or post-ictal state. According to his wife, the patient has been seizure-free since 2020 and is compliant with his medications. The patient denied chest pain, shortness of breath, dizziness, diaphoresis, history of substance abuse, or family history of ACS. He was alert and oriented to place, time, and person. His vital signs at this time were unremarkable; he was afebrile with a blood pressure of 110/74 mmHg and a heart rate of 63 bpm. Lab results demonstrated elevated HS-troponin to 151 ng/dL from 38 ng/dL the previous day, along with elevated lactic acid of 4.2 mmol/L. Pro-BNP was elevated to 1,705 pg/mL (Table 1). An EKG revealed normal sinus rhythm with S1Q3T3 morphology and T-wave inversions in V3-V5 (Figure 2). He was given an initial treatment of loading-dose aspirin and clopidogrel along with IV fluids and was admitted with an initial diagnosis of ACS.

**Table 1:** Pertinent lab tests from day 1 and day 2 of patient presentation BNP, brain natriuretic peptide; HS, high-sensitivity

Lab test	Value	Normal range	Time
HS-troponin	58.0 ng/mL	<14 ng/mL	Day 1
HS-troponin	38.0 ng/mL	<14 ng/mL	Day 1 repeat
Anion gap	19	4-12	Day 1
Creatinine	1.27 mg/dL	0.6-1.2 mg/dL	Day 1
HS-troponin	151.0 ng/dL	<14 ng/mL	Day 2
Lactic acid	4.2 mmol/L	0.5-2.2 mmol/L	Day 2
Pro-BNP	1705.0 pg/mL	<125 pg/mL	Day 2

**Figure 3:**
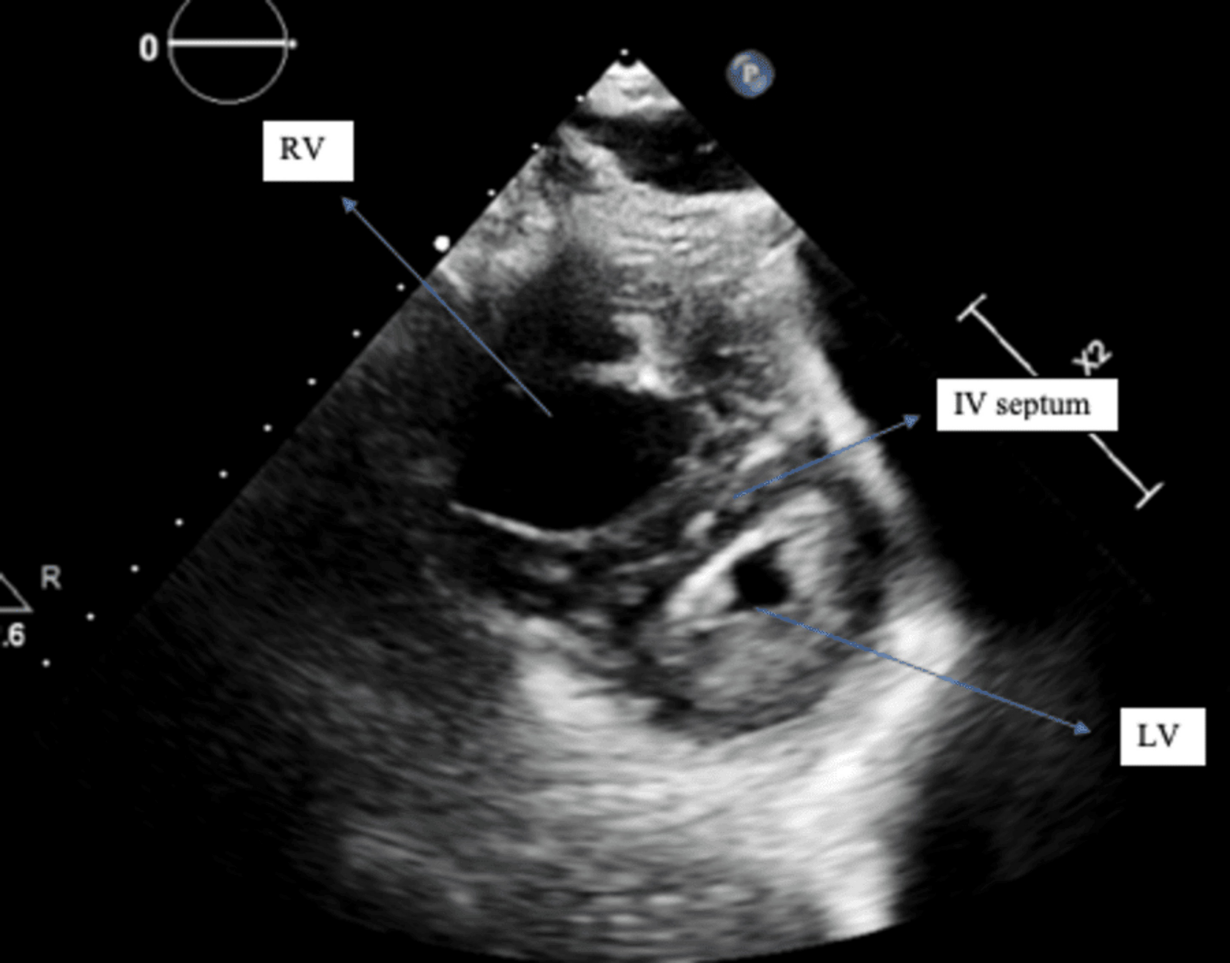
Transthoracic echocardiography (parasternal short axis view) showing severely dilated RV, small LV, and D-shaped interventricular septum. LV, left ventricle; RV, right ventricle

The patient underwent several diagnostic scans to evaluate the underlying cause. Chest X-ray was unremarkable. Computed tomography of the head was normal with no acute findings. Transthoracic echocardiography (TTE) showed an ejection fraction of 70% with D-shaped interventricular septum, suggestive of RV pressure and volume overload along with severely dilated RV and moderate RV dysfunction (Figure 3).

**Figure 4:**
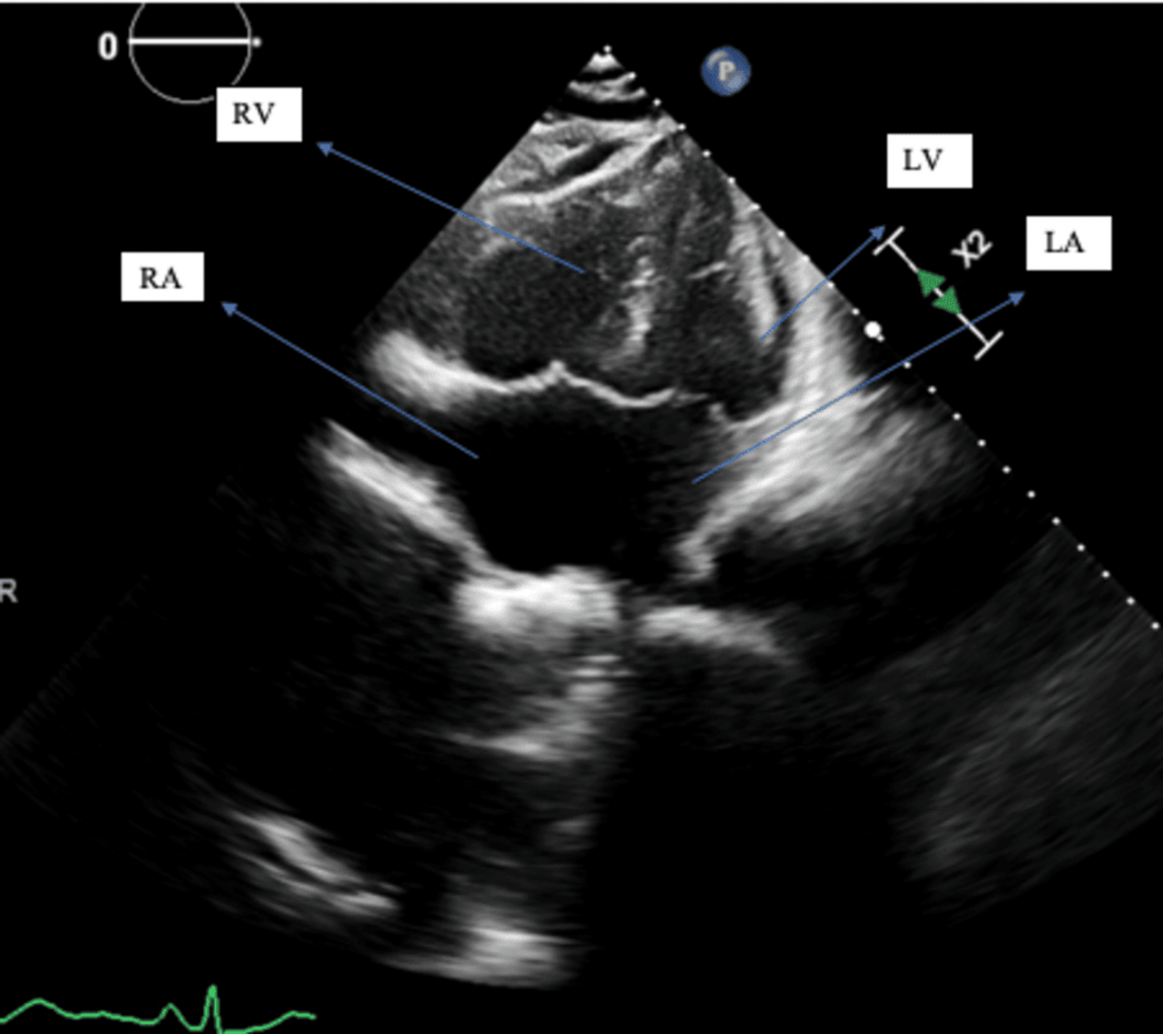
Transthoracic echocardiogram (apical four-chamber view) showing McConnell’s sign, which is akinesia of the right ventricular lateral wall with sparing of the apex. LA, left atrium; LV, left ventricle; RA, right atrium; RV, right ventricle

TTE was also significant for McConnell’s sign, which is akinesia of RV free wall with sparing of RV apex suggestive of PE (Figure 4).

**Figure 5:**
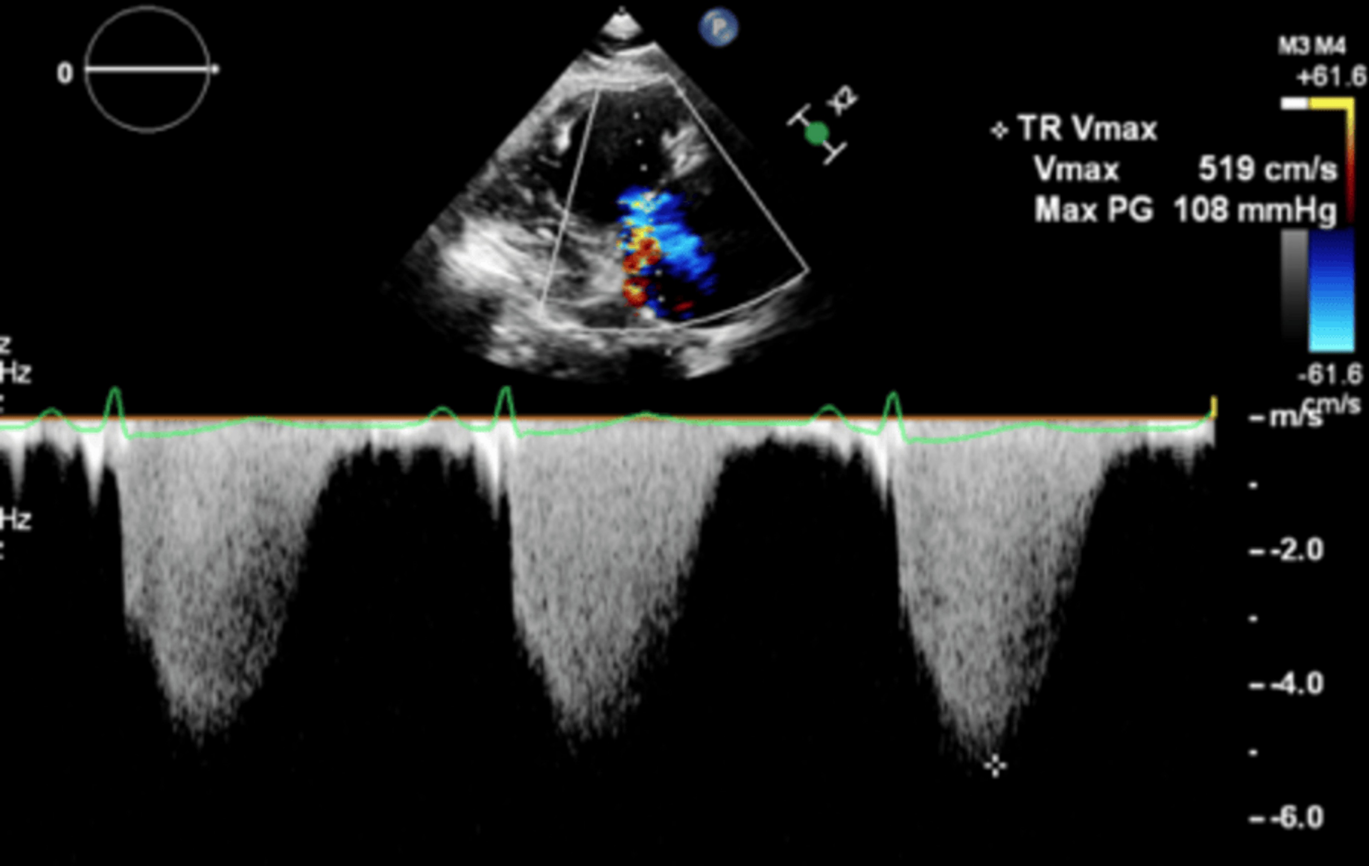
Transthoracic echocardiography showing the maximum velocity of the tricuspid regurgitation jet as 5.19 m/s and the maximum gradient as 108 mmHg. The RA pressure is 8 mmHg. The calculated PA systolic pressure is 116 mmHg (PASP = 4 X TRV2 + RA pressure). PA, pulmonary artery; PASP, pulmonary artery systolic pressure; RA, right atrium;TRV, tricuspid regurgitation velocity

Furthermore, severe pulmonary hypertension with a pulmonary artery systolic pressure of 116 mmHg was noted (Figure 5).

**Figure 6:**
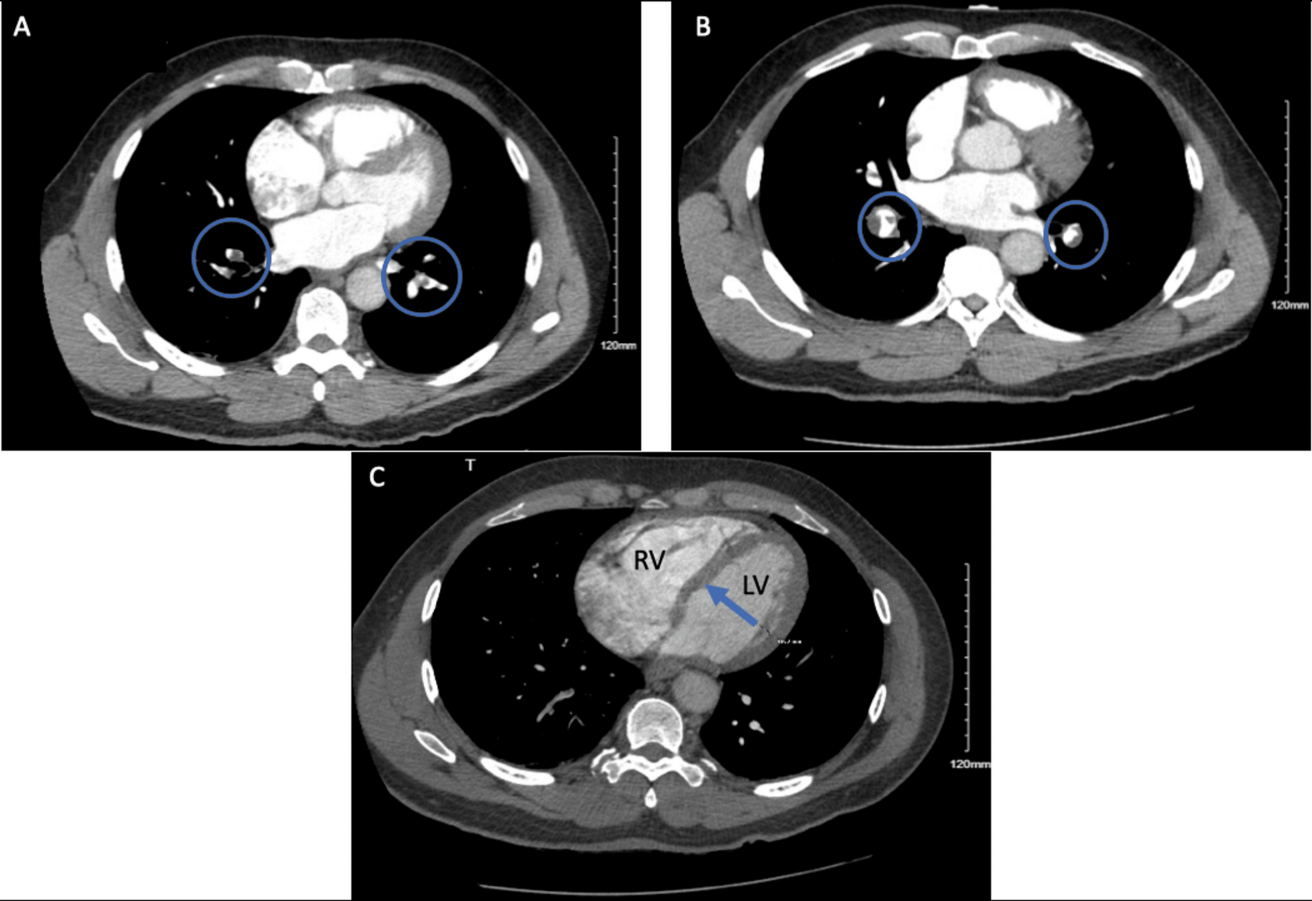
CTA showing bilateral filling defects signifying segmental and lobar pulmonary emboli (blue circles) in images A and B. Image C shows a flattened interventricular septum (blue arrow) with a relatively enlarged right ventricle signifying CTA evidence of right heart strain. CTA, computed tomography with angiography LV, left ventricle; RV, right ventricle

Computed tomography with angiography (CTA) further confirmed acute PE with bilateral interlobar segmental and proximal subsegmental pulmonary arteries with right heart strain (Figure 6). Lower extremity venous duplex was ordered, which was positive for acute deep vein thrombosis in bilateral femoral and popliteal veins. Repeat HS-troponin was 141 ng/mL. The patient was started on full-dose anticoagulation therapy as an inpatient. The patient's symptoms resolved, and a repeat TTE showed resolution of right-sided dilation and pulmonary hypertension.

## Discussion

ACS is defined by a reduction in blood supply to the heart with EKG changes including ST-segment elevated myocardial infarction (STEMI), non-STEMI, and unstable angina. More than one million people are hospitalized for ACS in the USA each year [6]. The most common presenting symptom of ACS is chest discomfort, although approximately 48% of women and 40% of men present with non-specific symptoms posing a diagnostic challenge [6]. Typically, in the emergency setting, cardiac troponin with EKG is used to diagnose and identify patients who require acute coronary angiography. In this case, the patient's troponin levels followed a pattern that raised suspicion for both ACS and PE. The initial presentation showed a peak troponin level of 58 ng/mL, which decreased to 38 ng/mL, suggesting a potential non-acute myocardial injury or a resolved ischemic event. However, on re-presentation, the troponin increased sharply to 151 ng/mL, indicating ongoing myocardial strain or injury. This marked rise in troponin could be attributed to the RV strain secondary to PE, as significant RV afterload increases lead to subendocardial ischemia [7]. In PE, troponin elevation is typically transient, peaking within hours of the embolic event due to RV ischemia but not demonstrating the same sustained elevation as seen in MI [7]. This distinction between the kinetics of troponin elevation in PE versus ACS underscores the importance of serial measurements in differentiating the two conditions. Echocardiography is a significant part of ACS work-up, which can reveal global or regional wall motion abnormalities and rule out effusion or valvular dysfunction. Despite it being not specific regarding the acuity of the disease, it plays an important role in patients with low pre-test likelihood of ACS, such as young age with absence of atherosclerotic risk factors and subjects presenting late after symptom onset [8,9]. This patient initially presented with symptoms resembling ACS, given the chest pain and rising cardiac troponin, but the diagnostic perspective quickly shifted following echocardiography findings.

In this case, the patient exhibited no signs or symptoms of PE on the first presentation and instead presented with elevated troponin levels and syncope that may be explained by ACS. The patient did not have tachycardia, tachypnea, hemoptysis, a history of malignancy, immobilization, recent surgery, or deep venous thrombosis. Given the patient’s presentation with syncope, a calculated Well’s score of 0 indicated a low pre-test probability for PE. The Well's score is a clinical prediction rule used to estimate the likelihood of PE based on factors such as clinical symptoms of DVT, heart rate, history of immobilization or recent surgery, and the absence of a more likely diagnosis. A score of 0-1 suggests a low probability of PE, thus typically not warranting an immediate CTA scan without additional risk factors [10]. This context influenced the initial diagnostic approach, where PE was deprioritized as a primary diagnosis, especially in the absence of other classic PE risk factors. Similarly, the patient did not present with hypertension and did not have associated back pain An aortic dissection was also lower on the differential diagnosis. Additionally, the patient decided against staying for observation for 24-48 hours on telemetry and chose to be discharged home; therefore, his syncopal episode was not completely worked up on the first presentation. The patient’s decision to leave the hospital against medical advice after his initial presentation posed a significant risk, as it curtailed further observation and diagnostic evaluation that could have clarified the etiology of his syncope. Observation on telemetry could have captured intermittent cardiac arrhythmias or further fluctuations in troponin levels, providing valuable clues regarding the patient's cardiac or pulmonary status. This missed opportunity for extended monitoring delayed a definitive diagnosis, emphasizing the challenges clinicians face when patients refuse recommended care.

On his second presentation, the patient had a troponin level that had quadrupled from the day before as well as had new changes on the EKG. The EKG findings played a pivotal role in shifting the diagnostic focus from ACS to PE. On the patient’s second presentation, the presence of T-wave inversions in V3-V5 and the S1Q3T3 pattern were noted. While the S1Q3T3 pattern - characterized by an S wave in lead I, a Q wave in lead III, and an inverted T wave in lead III - is a classic marker of right heart strain and is suggestive of PE, it is not highly sensitive, appearing in only around 20-50% of PE cases [11]. It has greater specificity when present, but its absence does not rule out the diagnosis. The T-wave inversions in the precordial leads (V3-V5) are more indicative of RV strain and have a higher sensitivity for PE than the S1Q3T3 pattern [7,11]. It has also been reported that an S1Q3T3 pattern is more commonly seen in PE when the troponin levels are increased [11]. These EKG changes, combined with the rising troponin and clinical presentation, were key in guiding further imaging with CTA, which confirmed the diagnosis of acute PE.

The diagnosis of PE can be diagnostically challenging given the nonspecific nature of clinical and lab findings. It can range from signs of paradoxical emboli to sudden cardiac death to an entirely asymptomatic presentation [12]. The incidence of first-time VTE is estimated at 70 to 113 cases per 100,000 per year. Due to asymptomatic and atypical presentation, it is believed that many cases of PE go unrecognized, with some investigators estimating that one to three cases remain unidentified [13]. A few clinical signs observed in PE are hypoxia, tachycardia, and tachypnea, which are often masked in intensive care unit patients requiring sedation and ventilation [13]. D-dimer testing is useful when PE or DVT is suspected with a negative predictive value of 95-98% in emergency settings [13]. Right heart dysfunction is seen on echocardiography in cases of massive and sub-massive PE; however, the gold standard for PE diagnosis is CTA for confirmatory results [14]. Severe untreated PE can result in circulatory failure and sudden death, highlighting the critical importance of timely and accurate diagnosis and treatment [14]. PE can lead to syncope, but this is an atypical finding. The compression of the left ventricle secondary to a dilated right ventricle can lead to reduced pre-load and low cardiac output, therefore causing syncope. Consequently, echocardiography became a crucial diagnostic tool not only for identifying PE but also for clarifying the patient’s condition.

The diagnostic accuracy of TTE in diagnosing PE was studied through a systematic review and meta-analysis of PubMed, CINAHL, and EMBASE in 2016 [15]. Right heart strain was the most commonly identified sign on echocardiography with a sensitivity of 53% and specificity of 83% [15]. The 60/60 sign, a specific marker for diagnosing PE, was also observed, which is pulmonary acceleration time under 60 ms and a tricuspid regurgitation jet gradient under 60 mmHg [16]. Additionally, several other distinct signs were identified, including decreased RV free wall motion and systolic function, RV dilatation, morphology and motion of interventricular septum, ventricle size ratio, abnormal septal motion, McConnell’s sign, right heart thrombus, RV hypokinesis, and pulmonary hypertension. The conclusion drawn was that echocardiography showed consistently high specificity, making it a crucial rule-in test in critical care settings, particularly those patients who cannot undergo other confirmatory tests [16]. McConnell’s sign is defined as regional RV dysfunction with akinesia of the mid-free wall with normal motion at the apex, which is a well-known finding in the setting of acute PE [17,18]. In a cohort study of 126 patients, McConnell et.al. first described this sign in 1996, using it to aid in diagnosing acute PE. It indicates a significant pulmonary perfusion defect and severe hemodynamic instability that can lead to death, thus contributing to early decision-making regarding the use of thrombolysis [17].

Understanding the similarities in clinical signs and presentations between ACS and PE is crucial for making an accurate diagnosis. In massive PE, elevated troponin levels were observed in 50% of patients, in sub-massive PE, they were observed in 35%, and in non-massive PE, they were observed only in isolated cases [19]. The rise in troponin occurs due to subendothelial ischemia in RV due to increased RV strain [19]. The kinetics of cardiac troponin concentration in PE are different from those in ACS, indicating distinct mechanisms of cardiomyocyte injury and troponin elevation. In a study, 56% of patients with acute PE had positive troponin T levels that peaked at a median of 0.48 μg/L after 10 hours and remained elevated for 30-40 hours, whereas troponin levels in MI rose more gradually to median concentrations of 0.22-0.41 μg/L, with fluctuating peaks and troughs, and stayed elevated for over 120 hours [19]. The case presented also showed a similar pattern of troponin elevation, as seen in PE patients. Chest pain, tachycardia, and tachypnea are common clinical symptoms shared by both ACS and PE, making differentiation challenging. Echocardiography is a crucial tool for rapidly distinguishing between these conditions, as it provides immediate insights into the underlying pathology. Given the acute nature of both diseases and the need for prompt treatment, echocardiographic findings can help direct the diagnosis accurately and initiate appropriate management without delay.

## Conclusions

Echocardiography can be a crucial tool in distinguishing between PE and ACS. Its high specificity makes it an effective initial diagnostic tool for suspected PE, particularly in patients with a suitable risk profile. This is especially beneficial for critically ill patients in the ED or intensive care unit who cannot undergo other confirmatory tests. Additionally, echocardiography helps to rule in or rule out ACS in cases with atypical, non-specific presentations, facilitating timely diagnosis and reducing mortality and morbidity.
